# Does stunting modify the impact of salivary factors and early childhood caries on oral health–related quality of life?

**DOI:** 10.1186/s12889-026-27304-5

**Published:** 2026-05-07

**Authors:** Arlette Suzy Setiawan, Meitty Caesarini, Iwan Ahmad Musnamirwan, Risti Saptarini Primarti, Ratna Indriyanti

**Affiliations:** 1 Department of Pediatric Dentistry, Faculty of Dentistry, Universitas Padjadjaran, Bandung, Indonesia; 2 Pediatric Dentistry Residency Program, Faculty of Dentistry, Universitas Padjadjaran, Bandung, Indonesia

**Keywords:** Child, Preschool, Dental Caries, Saliva, Hydrogen-Ion Concentration, Quality of Life, Growth Disorders

## Abstract

**Background:**

Stunting, a form of chronic malnutrition, is associated with impaired growth and potential changes in salivary function that may increase susceptibility to early childhood caries (ECC). These oral conditions may further influence a child’s oral health–related quality of life (OHRQoL). However, the extent to which stunting modifies the impact of salivary factors and ECC on OHRQoL remains unclear.

**Methods:**

This analytical cross-sectional study included 210 preschool children aged 24–59 months in Bandung, Indonesia, equally divided into stunted and non-stunted groups based on height-for-age z-scores (HAZ <-2 SD for stunted). Dental caries was assessed using the deft index, while salivary pH and buffering capacity were measured using the GC Saliva Check Buffer Test. OHRQoL was evaluated using a modified Early Childhood Oral Health Impact Scale (ECOHIS) with a dichotomous response format (*never = 0*,* ever = 1*) validated through pilot testing (Cronbach’s α = 0.870). Statistical analyses included Mann–Whitney U, Chi-square, and t-tests, followed by logistic regression with interaction terms to examine effect modification by stunting.

**Results:**

Non-stunted children had higher mean deft scores (6.50 ± 4.43) compared to stunted children (5.12 ± 4.38, *p* = 0.020). Acidic Saliva was more frequent among stunted children (11.4%) than non-stunted children (3.8%, *p* = 0.037). However, no significant differences were observed in ECOHIS total scores or in the impact of OHRQoL between groups (*p* > 0.05). Logistic regression revealed no significant interactions between stunting, ECC, salivary pH, or buffering capacity (all *p* > 0.05).

**Conclusion:**

Stunting did not affect the association between salivary factors, early childhood caries, and oral health-related quality of life in preschool children. Although children with stunted had slightly higher salivary acidity, these differences did not result in measurable differences in quality of life. Prevention should combine oral health education and nutritional interventions to comprehensively address the risk of stunting and dental caries.

## Introduction

Early childhood is a critical “golden period” in human development, characterized by rapid physical, cognitive, and emotional growth [1]. During this period, optimal nutrition determines whether children can reach their full developmental potential or suffer from growth faltering, such as stunting - a form of chronic malnutrition [2]. The World Health Organisation defines stunting as a condition in which a child of given age is more than two standard deviation below the WHO median growth rate for children. This reflects long-term nutritional deficiencies and recurrent infections [3]. Stunting remains a significant public health problem globally, including in Indonesia. In 2023, the national prevalence of stunting in Indonesia reached 21.5%, consistently exceeding the WHO threshold of 20% [4]. In Bandung, 16.3% of children under five years of age were affected by stunting in 2023, highlighting the need for targeted intervention [5].

In addition to its systemic consequences, stunting can alter oral physiology, particularly the composition and function of saliva [6]. Studies have found that malnourished children have reduced saliva flow, lower pH levels, and decreased buffering capacity, which may make them more susceptible to early childhood caries (ECC) [6, 7]. Saliva plays a vital role in maintaining oral homeostasis, neutralizing acidic substances and promoting tooth remineralization. Therefore, alterations in saliva properties can accelerate tooth demineralization, thus promoting the progression of caries [8, 9].

Previous studies have shown that children without caries typically have higher saliva pH and buffering capacity than children with early caries. This suggests that saliva quality influences susceptibility to caries [8]. In stunted children, chronic malnutrition impairs salivary gland function, reducing protective components and increasing the risk of caries [10].

Early childhood caries is one of the most common chronic diseases in young children worldwide, with up to 84.9% of children under five years old in Indonesia suffering from it [11, 12]. ECC can lead to pain, infection, tooth loss, and difficulty chewing, ultimately impacting children’s nutrition, growth and development, and mental health [13]. These effects extend beyond physical symptoms to the emotional and social aspects of children’s daily lives—collectively known as oral health-related quality of life (OHRQoL) [14].

OHRQoL is a multidimensional concept that reflects how oral health problems affect children’s and their families’ comfort, self-esteem, social interaction, and overall well-being [15]. Previous studies have shown that higher caries prevalence and reduced saliva production are associated with lower OHRQoL [16, 17]; however, the extent to which stunting alters this relationship remains unclear.

To date, few studies have explored whether biological changes associated with growth retardation enhance or diminish the effects of oral health problems on OHRQoL [6, 18].

Understanding this interaction is crucial for developing preventative and integrated strategies that take into account both nutrition and oral health in early childhood. Therefore, the present study aimed to investigate whether stunting modifies the relationship between salivary characteristics (pH and buffering capacity), early childhood caries, and oral health–related quality of life (OHRQoL) among preschool children.

## Material and method

### Study design and participants

This analytical observational study employed a cross-sectional design and was conducted from January to June 2025 in Bandung, Indonesia. The study population comprised 182 stunted children aged 12–60 months registered at integrated health posts (Posyandu) in four priority stunting areas designated by the Bandung City Government: Sukagalih, Antapani Tengah, Dago and Sekeloa subdistricts [19]. These areas were selected because they represent urban communities with diverse socioeconomic conditions and have been identified as priority loci for stunting reduction programs [20].

Based on previous studies reporting differences in dental caries prevalence between stunted and non-stunted children [18, 21], a two-proportion comparison formula was used to estimate the minimum required sample size. With a statistical power of 80% (β = 0.20), a significance level of 5% (α = 0.05), and an expected proportion difference of 20%, the minimum sample size per group was calculated to be 95 participants. To compensate for potential dropouts or incomplete data, a 10% margin was added, resulting in a final target sample size of approximately 105 children per group. Therefore, the analysis included a total of 210 participants.

Inclusion criteria were children aged 12–60 months, cooperative during dental examination (children were considered cooperative if they were able to undergo a brief oral examination without excessive distress, crying, or refusal that prevented completion of the dental assessment). Children who were unable to tolerate the examination were excluded from the study. In addition, children with systemic diseases, congenital abnormalities, or undergoing orthodontic treatment were also excluded.

From this population, 105 stunted children who met the inclusion criteria were recruited using consecutive sampling during routine Posyandu health monitoring visits.

An additional 105 non-stunted children, matched by age range (12–60 months) and sex distribution, were subsequently selected from the same locations to serve as the comparison (control) group.

Based on anthropometric data, participants were divided into two groups: a growth retardation group (*n* = 105) and a normal growth group (*n* = 105). Growth retardation was defined according to the World Health Organization’s child growth standards, i.e., a height-age Z-score (HAZ) ≤ -2 SD, while normal growth children have an HAZ score > -2 SD [21].

### Ethical considerations

The study protocol was approved by the Research Ethics Committee, Universitas Padjadjaran (Approval No. 125/UN6.KEP/EC/2025). Written informed consent was obtained from parents or legal guardians prior to participation.

### Data collection procedures

1. Anthropometric measurement

Body height was measured using a calibrated stadiometer with a precision of 0.1-cm while the children stood upright without shoes. Age was obtained from birth records or parental report. Using the WHO-Anthro software [22], height-specific Z-scores (HAZ) were calculated based on the World Health Organization’s child growth criteria. Children with HAZ < -2 standard deviations (SD) were classified as stunting, and children with HAZ > -2 SD were classified as developing normally (non-stunting) [23]. This classification was intended to determine whether a child was fit to be included in the underdeveloped group and to identify age- and sex-matched developing normally children from the same population.

2. Dental examination

Dental status was assessed by calibrated examiners under natural light using mouth mirrors and sterile probes. Caries experience was recorded using the decayed, extracted, and filled teeth (deft) index according to WHO Oral Health Surveys – Basic Methods [24].

Prior to data collection, the examiners underwent a calibration exercise using 20 children who were not included in the main study sample. The inter-examiner reliability was assessed using Cohen’s kappa statistic [25], yielding a kappa value of 0.81, indicating excellent agreement.

In this index, the “extracted” (e) component included teeth that had been removed or presented as root remnants due to caries destruction, while teeth missing because of physiologic exfoliation were excluded.

For analytical purposes, ECC status was derived from the deft index and lychotomous: children with deft > 0 were categorized as having ECC (ECC present), whereas those with deft = 0 were classified as caries-free. This classification was used for the main comparative analyses.

Second, the severity of ECC was further categorized to describe the distribution of disease within the study population. Based on the number of affected primary teeth recorded in the deft index, ECC severity was classified as free caries (deft = 0), mild ECC (1–3 affected teeth), moderate ECC (4–6 affected teeth), and severe ECC (*≥* 7 affected teeth) [26].

3. Salivary analysis

Unstimulated Saliva was collected between 09:00–11:00 a.m. at least 1 h after the last food intake. Salivary pH and buffer capacity were measured using GC Saliva Check Buffer Test (GC Corporation, Tokyo, Japan) according to the manufacturer’s instructions.

Salivary acidity was classified as *normal* (pH ≥ 6.2) or *acidic* (pH < 6.2) [27].

4. Oral Health-Related Quality of Life (OHRQoL)

Oral health-related quality of life was assessed using the Early Childhood Oral Health Impact Scale (ECOHIS), a validated questionnaire designed to evaluate the impact of oral health condition on preschool children and their families. The ECOHIS scale comprises 13 items, divided into two parts: the “Impact on Children” Sect. (9 items) assesses symptoms, function, psychological status, self-image, and social interaction; and the “Impact on Families” Sect. (4 items) assesses parental burden and family functioning [28].

To improve comprehension among respondents with varying levels of education, the original 5-point Likert scale (never, almost never, occasionally, often, very often) was simplified to a dichotomous response format (never = 0; ever = 1). This adjustment was made because pilot studies showed that some parents had difficulty distinguishing between the middle options. The simplified dichotomous rating has also been applied in population-based studies to improve the reliability of responses and minimize measurement error in lower-education groups [29].

The ECOHIS total score is calculated by summing the scores of all items, thus ranging from 0 to 13 points, with higher scores indicating a greater negative impact on oral health-related quality of life [28]. In the analysis, the total score was divided into an impacted group (score > 1) and an non-impacted group (score = 0). Before data collection, the modified ECOHIS questionnaire was tested on 30 parents, and the results showed that it had good internal consistency with a Cronbach’s alpha coefficient of 0.870 [30].

### Statistical analysis

The data were analyzed using IBM SPSS Statistics version 23.0. Descriptive statistics (mean ± standard deviation, median [interquartile range], and frequency [percentage]) were used to summarize the characteristics of the participants.

Normality was tested using the Shapiro–Wilk test [31].

- For normally distributed variables, comparisons between stunted and non-stunted groups used the independent t-test.
- For non-normally distributed variables, the Mann–Whitney U test was applied.
- Chi-square tests were used for categorical variables. All p-values < 0.05 were considered statistically significant.

To examine effect modification, a binary logistic regression model was constructed with ECOHIS impact (0 = unimpacted, 1 = impacted) as the dependent variable.

Predictor variables included ECC status, salivary pH, buffer capacity, and nutritional status, with interaction terms (ECC × stunting, pH × stunting, buffer × stunting).

Model fit was evaluated using the Hosmer–Lemeshow goodness-of-fit test, Nagelkerke R², and overall classification accuracy.

## Result

### Participant characteristics

A total of 210 children aged 12–60 months were included in the analysis, consisting of 105 non-stunted and 105 stunted participants. The characteristics of the study participants are shown in Table 1.

**Table 1:** Characteristics of the Participants According to Nutritional Status (Stunting vs. non-stunting)

Characteristics	Non-Stunting (*n* = 105)	Stunting (*n* = 105)	
Continuous variables			
Age (month), mean *±* SD	35.10 *±* 12.703	35.39 *±* 12.878	0.879
def-t, mean *±* SD	6.50 *±* 4.427	5.12 *±* 4.380	0.020*
def-t%, mean *±* SD	32.67 *±* 21.925	26.59 *±* 21.733	0.054
pH Saliva, mean *±* SD	6.488 *±* 0.2601	6.436 *±* 0.3111	0.195
Buffer Capacity, mean *±* SD	3.43 *±* 1.580	3.00 *±* 1.787	0.071
Total ECOHIS, median (IQR)	4	4	0.691
- Child impact	3	3	0.887
- Family impact	1	1	0.084
Categorical variables			
Gender, n (%)			
- Male	53 (50.5%)	53 (50.5%)	
- Female	52 (49.5%)	52 (49.5%)	
ECC Status, n (%)			0.012*
- ECC present	98 (93.3%)	86 (81.9%)	
- ECC absent	7 (6.7%)	19 (18.1%)	
Type of ECC, n (5)			0.088
- Free caries	7 (6.7%)	19 (18.1%)	
- mild	24 (22.9%)	19 (18.1%)	
- moderate	25 (23.8%)	24 (22.9%)	
- severe	49 (46.7%)	43 (41.0%)	
Saliva acidity, n (%)			0.037*
- normal	101 (96.2%)	93 (88.6%)	
- acid	4 (3.8%)	12 (11.4%)	
OHRQoL impact, n (%)			0.051
- impacted	67 (63.8%)	53 (50.5%)	
- un-impacted	38 (36.2%)	52 (49.5%)	

Continuous variables are presented as median (interquartile range) for non-normally distributed data and mean ± SD for normally distributed data. p-values were obtained using Mann–Whitney U tests for non-parametric data, independent t-tests for normally distributed data, and Chi-square or Fisher’s exact tests for categorical data. Bold values indicate statistical significance at *p* < 0.05

The sex distribution of the two groups was balanced, with 53 boys (50.5%) and 52 girls (49.5%) in each group. The mean age of the participants in the two groups was similar (35.10 ± 12.70 months in the group non-stunting and 35.39 ± 12.88 months in the group with stunting).

Regarding dental status, the mean deft score was slightly higher in non-stunted children (6.50 ± 4.43) than in those who were stunted (5.12 ± 4.38). Similarly, the mean deft % was 32.67 ± 21.93 in non-stunted and 26.59 ± 21.73 in stunted children. Most participants in both groups had early childhood caries (ECC), with a higher prevalence among non-stunted (93.3%) compared to stunted children (81.9%). Across ECC severity, the severe type predominated (46.7% in non-stunted vs. 41.0% in stunted).

Salivary findings showed that the mean pH was within the normal range for both groups (6.49 ± 0.26 vs. 6.44 ± 0.31). However, acidic saliva was observed more frequently among stunted children (11.4%) than non-stunted (3.8%). The mean buffer capacity was slightly lower in the stunted group (3.00 ± 1.79) than in the non-stunted group (3.43 ± 1.58).

For oral-health-related quality of life, both groups had similar median total ECOHIS scores (4, IQR = 1–7). The child impact domain contributed more to the overall score (median = 3) compared to the family impact domain (median = 1). A higher proportion of non-stunted children experienced an ECOHIS impact (63.8%) than stunted children (50.5%), although the difference was not marked.

### Bivariate analysis of nutritional status and oral health indicators

Comparative analysis between stunted and non-stunted children is presented in Table 1. No significant difference was observed in age distribution between groups (*p* = 0.879). The mean deft score was significantly higher among non-stunted children compared to stunted children (*p* = 0.020), while the difference in deft percentage approached but did not reach statistical significance (*p* = 0.054).

The salivary pH was similar in both groups (6.49 ± 0.26 vs. 6.44 ± 0.31; *p* = 0.195).

The buffer capacity was slightly higher in non-stunted children, but the difference was not statistically significant (*p* = 0.071). Likewise, the median total ECOHIS score, as well as its child and family subdomains, did not differ significantly between stunted and non-stunted participants (all *p* > 0.05).

In contrast, ECC prevalence was significantly higher among non-stunted children (93.3%) compared to their stunted counterparts (81.9%; *p* = 0.012).

Although the distribution of ECC severity types (mild, moderate, severe) showed no significant difference between groups (*p* = 0.088), a greater proportion of non-stunted children presented with the severe type. Furthermore, acidic saliva was found more frequently among stunted children (11.4%) than among non-stunted ones (3.8%), and this difference was statistically significant (*p* = 0.037).

Regarding oral health–related quality of life, a higher proportion of non-stunted children reported an ECOHIS impact(63.8%) compared to stunted children (50.5%), but the difference was not statistically significant (*p* = 0.051).

### Multivariable analysis with interaction terms

Binary logistic regression analysis was performed to identify factors associated with oral health–related quality of life (OHRQoL) impact, measured using the ECOHIS instrument, and to examine potential effect modification by nutritional status.

As shown in Table 2, none of the main predictors—ECC status, salivary pH, buffer capacity, or nutritional status—were significantly associated with OHRQoL impact (all *p* > 0.05).

**Table 2:** Logistic Regression with Interaction Terms Showing Effect Modification on Oral Health-Related Quality of Life Impact

Variables	B	S.E	Wald	OR (Exp B)	95% CI for OR	*p*-value
ECC status (Ref: caries-free)	-0.301	0.793	0.145	0.74	0.16–3.50	0.704
pH Saliva	-0.293	0.790	0.137	0.75	0.16–351	0.711
Nutritional status (Ref: non-stunted)	-0.372	6.886	0.003	0.69	0.00-5.01 × 10^5^	0.957
ECC x Stunting	1.082	0.984	1.209	2.95	0.43–20.27	0.271
pH x Stunting	-0.376	1.076	0.122	0.69	0.08–5.66	0.727
Buffer x Stunting	0.205	0.126	2.628	1.23	0.96	0.105
Constant	2.859	4.606	0.385	-	-	0.535

Binary logistic regression was performed to identify predictors of oral health-related quality of life impact (ECOHIS). Interaction terms were included to test for effect modification by nutritional status. Odds ratios (OR) and 95% confidence intervals (CI) are presented. Bold values indicate statistical significance at *p* < 0.05. Reference categories: caries-free for ECC status and non-stunted for nutritional status

Interaction terms were introduced to test for effect modification by stunting.

The interactions ECC status × stunting, pH × stunting, and buffer × stunting were not statistically significant, indicating that nutritional status did not modify the relationship between these oral health parameters and OHRQoL impact.

The logistic regression model demonstrated a good overall fit (Hosmer–Lemeshow χ² = 2.80, *p* = 0.903) and explained approximately 7.8% of the variance in OHRQoL impact (Nagelkerke R² = 0.078), with an overall classification accuracy of 60.5%.

A comparison of ECOHIS domain scores between stunted and non-stunted children is presented in Table 3. No statistically significant differences were observed between the two groups in terms of total ECOHIS score, impact on children, or impact on families (*p* > 0.05).

**Table 3:** Comparison of ECOHIS Domain Between Stunted and Non-Stunted Children

ECOHIS Domain	Non-stunted (*n* = 105)	Stunted (*n* = 105)	*p*-value
Total ECOHIS score	1 (0–4)	1 (0–4)	0.691
Child impact	1 (0–3)	1 (0–3)	0.887
Family impact	0 (0–1)	0 (0–1)	0.084

Values are presented as median (interquartile range)

*p*-values were obtained using Mann-Whitney U test

Secondary descriptive findings showed that the child impact domain contributed the largest proportion of ECOHIS scores in both groups, while family impact scores were generally low. The distribution of ECC severity was similar between groups (*p* = 0.088).

## Discussion

This study examined the relationship between nutritional status, early childhood caries (ECC), salivary characteristics, and oral health–related quality of life (OHRQoL) among children under five years old. Although children with stunting are often presumed to have a higher risk of oral health problems due to compromised growth and micronutrient deficiency, the present findings showed that nutritional status alone was not a significant predictor of OHRQoL.

Regardless of the presence or absence of stunting, the prevalence of early childhood caries (ECC) was high, with similar ECOHIS scores and caries area. This suggests that, in addition to the potential biological susceptibility associated with stunting, sociopsychological and behavioral factors significantly influence children’s perception of the impact of oral health on daily life.

Studies have found that children without growth retardation had higher deft scores and a higher prevalence of ECC compared to their age-matched peers. This finding is consistent with previous studies on preschool children in Indonesia, which reported a persistently high caries burden across all dietary groups [32, 33]. Similar trends have been observed in other low- and middle-income countries, where caries distribution was more correlated with sugar intake, oral hygiene behaviors, and parental supervision than with anthropometric indicators [34–37].

Socioeconomic and environmental factors may also contribute to differences in childhood caries risk. Previous research has shown that family income, living conditions, and access to resources influence children’s oral health. For example, Nurelhuda et al. found that school-aged children of higher socioeconomic status in Sudan were reported to be at high risk of dental caries, highlighting the complex interrelationship between socioeconomic status and oral health behaviors [38]. These findings suggest that dental caries risk is not solely determined by biological or nutritional factors, but is also influenced by dietary habits, parental awareness, and access to preventive dental care.

Therefore, these results reinforce the view that the burden of early childhood caries is a pervasive public health issue affecting all dietary categories, and that interventions should prioritize preventive behaviors and parental education rather than solely focusing on growth indicators [39, 40].

### Salivary parameters and oral health-related quality of life

Salivary properties, including pH and buffering capacity, play a crucial role in maintaining oral ecosystem balance and modulating dental caries risk in young children [41].

In this study, both parameters were within physiological ranges in both malnourished and normally developing children, and no significant correlation was observed with oral health-related quality of life outcomes. This suggests that, despite differences in metabolism during growth and development, the functions of saliva in neutralizing acid and protecting tooth enamel are largely preserved.

Previous studies have focused on the relationship between salivary parameters and the incidence of dental caries, but the results on the role of pH and buffering capacity in predicting early childhood caries (ECC) are inconsistent [41]. At the same time, studies on preschool children have shown that OHRQoL is more strongly influenced by caries severity, pain, and functional limitations than by underlying biological parameters [42, 43]. To our literature research, no previous study has directly evaluated the association between salivary pH or buffering capacity and OHRQoL using ECOHIS in young children [44–46]; therefore the present findings add novel evidence suggesting that variations in basic salivary physicochemical properties may not necessarily translate into perceived quality of life impacts.

Interestingly, the proportion of children with acidic saliva was slightly higher among malnourished children, which may reflect dietary habits (e.g., increased carbohydrate intake or impaired chewing function leading to reduced saliva production) rather than systemic malnutrition itself [47]. Although this trend did not significantly affect ECOHIS scores, it highlights the potential importance of saliva analysis as an early functional biomarker in malnourished populations. Previous study has shown that cariogenic food intake can acutely reduce salivary pH in children [48] and that stunted toddlers may have lower saliva buffering capacity than their normal counterparts [49].

Future studies integrating salivary metabolomics or microbiome profiling could clarify whether subclinical biochemical alterations in saliva precede observable impacts on oral health–related quality of life. The lack of a significant correlation between salivary pH or buffering capacity and ECOHIS scores highlights the multifactorial nature of oral health-related quality of life (OHRQoL).

Children’s discomfort and parents’ anxiety (measured across ECOHIS domains) may be more sensitive to obvious symptoms such as pain, feeding difficulties, or sleep disturbances than to subtle physiological changes in salivary composition [50, 51].

This further underscores the need for integrated behavioral and parent education programs, as psychosocial factors may have a greater impact on perceived OHRQoL than purely biochemical factors.

Despite the contributions of this study, several limitations remain. First, the cross-sectional study design cannot infer a causal relationship between nutritional status and oral health. Second, salivary characteristics were assessed through simple tests performed directly in the dental chair, rather than comprehensive biochemical or microbiological analyses, which may have limited sensitivity to subtle changes. Third, the use of parent-reported ECOHIS questionnaire results carries the risk of recall bias and social expectation bias.

Another limitation of this study is the lack of a comprehensive assessment of dietary habits and oral hygiene behaviors. These factors are known to play a significant role in the development and progression of dental caries and may influence oral health-related quality of life. Future research, with detailed investigations into dietary habits, sugar intake, and brushing behaviors, will contribute to a more comprehensive understanding of the relationship between nutrition, oral health, and quality of life in early childhood.

Finally, this study was conducted only in a single regional population, which may limit the applicability of its results to other regions with different dietary patterns or access to healthcare resources. Future research should include longitudinal, multicenter studies incorporating salivary biomarkers, microbiome profiling, and behavioral assessments to elucidate the complex interrelationships between nutrition, oral health, and quality of life in early childhood.

From a public health perspective, these findings have significant implications for child health programs in Bandung and similar urban areas. The high prevalence of early caries (ECC) in both stunting and non-stunting children indicates that oral health problems are widespread and should not be viewed solely from a nutritional status perspective. Therefore, prevention strategies should be integrated into existing community-based child healthcare services, such as the child growth monitoring service already implemented in Posyandu. Incorporating early oral health screenings, sugar intake counseling for parents, and guidance on proper brushing techniques into stunting prevention programs can help reduce the burden of early childhood caries and improve children’s overall health. Such integrated approaches could strengthen current public health initiatives targeting early childhood development.

## Conclusion

In conclusion, this study found that stunting did not modify the relationship between salivary factors, early childhood caries, and oral health–related quality of life among preschool children. Although salivary acidity and buffer capacity differed slightly by nutritional status, these variations did not translate into differences in ECOHIS scores or perceived quality-of-life impacts.

The study results indicate that the impact of saliva quality and dental caries on children’s daily functioning and health is independent of their growth and development. Therefore, interventions aimed at improving oral health-related quality of life (OHRQoL) should prioritize behavioral and preventative measures, such as oral hygiene education, nutritional counseling, and early dental checkups, rather than focusing solely on improving nutrition.

## Data Availability

The datasets used and/or analysed during the current study are available from the corresponding author, Prof Arlette Suzy Setiawan (email: arlette.puspa@unpad.ac.id), on reasonable request.

## Abbreviations

OHRQoL: Oral Health–Related Quality of Life
OHIS: Oral Hygiene Index Simplified
def-t: decayed, extracted, and filled teeth (primary)
SD: Standard Deviation
